# Innovative Rocuronium Bromide Topical Formulation for Targeted Skin Drug Delivery: Design, Comprehensive Characterization, In Vitro 2D/3D Human Cell Culture and Permeation

**DOI:** 10.3390/ijms24108776

**Published:** 2023-05-15

**Authors:** Victor H. Ruiz, David Encinas-Basurto, Bo Sun, Basanth Babu Eedara, Eunmiri Roh, Neftali Ortega Alarcon, Clara Curiel-Lewandrowski, Ann M. Bode, Heidi M. Mansour

**Affiliations:** 1 Skaggs Pharmaceutical Sciences Center, College of Pharmacy, The University of Arizona, Tucson, AZ 85721, USA; 2 Department of Physics, Mathematics and Engineering, Campus Navojoa, Universidad de Sonora, Sonora 85880, Mexico; 3 The University of Arizona Cancer Center, Skin Cancer Institute, Tucson, AZ 85721, USA; 4 Center for Translational Science, Florida International University, Port St. Lucie, FL 34987, USA; 5 The Hormel Institute, University of Minnesota, Austin, MN 55912, USA; 6 Department of Cosmetic Science, Kwangju Women’s University, Gwangju 62396, Republic of Korea; 7 University of Arizona Cancer Center, University of Arizona, Tucson, AZ 85724, USA; 8 Department of Medicine, Division of Dermatology, The University of Arizona College of Medicine, Tucson, AZ 85724, USA; 9 Department of Medicine, Division of Translational & Regenerative Medicine, College of Medicine, The University of Arizona, Tucson, AZ 85724, USA; 10 BIO5 Institute, The University of Arizona, Tucson, AZ 85721, USA

**Keywords:** drug flux, Franz cell diffusion system, Strat-M^®^ synthetic biomimetic membrane, HaCaT human skin cell line, NHEK human primary skin cells, EpiDerm^®^ 3D human skin tissue

## Abstract

Cutaneous squamous cell carcinoma (cSCC) is the second-most common type of non-melanoma skin cancer and is linked to long-term exposure to ultraviolet (UV) radiation from the sun. Rocuronium bromide (RocBr) is an FDA-approved drug that targets p53-related protein kinase (PRPK) that inhibits the development of UV-induced cSCC. This study aimed to investigate the physicochemical properties and in vitro behavior of RocBr. Techniques such as thermal analysis, electron microscopy, spectroscopy and in vitro assays were used to characterize RocBr. A topical oil/water emulsion lotion formulation of RocBr was successfully developed and evaluated. The in vitro permeation behavior of RocBr from its lotion formulation was quantified with Strat-M^®^ synthetic biomimetic membrane and EpiDerm™ 3D human skin tissue. Significant membrane retention of RocBr drug was evident and more retention was obtained with the lotion formulation compared with the solution. This is the first systematic and comprehensive study to report these findings.

## 1. Introduction

The incidence of cutaneous squamous cell carcinoma (cSCC), a non-melanoma skin cancer, rises 2–4% per year in the United States [1]. A significant challenge in estimating accurate incidence rate of cSCC is the lack of reliable registries for non-melanoma skin cancer. Recent reports indicate that, if we apply the increase in incidences seen when multiple cSCCs per patient are included with the first cSCC, it is possible that the incidences could be as large as 112.8 per 100,000 person-years for male patients and 92.7 per 100,000 person-years for female patients. These incidences would be greater than the incidence of prostate cancer in males and approach the incidence of breast cancer in females in the US [2,3].

In addition, health care costs and morbidity associated with skin cancer have continued to increase in recent years [4]. Therefore, development of effective, preventive and therapeutic techniques is imperative. Ultraviolet (UV) radiation from the sun (sUV) is a significant environmental carcinogen that induces inflammation and skin cancer. Long-term exposure to sUV can trigger inflammatory reactions, oxidative stress, DNA damage and gene alterations in the skin, which have been linked to various skin conditions, including an increased risk of skin cancers [5]. Hence, identifying the primary signaling molecules involved in cSCC development for more precise treatments would be advantageous. Roh, Lee et al. have reported that the T-LAK cell-originated protein kinase (TOPK) and p53-related protein kinase (PRPK) are critical players in the development of skin malignancy and that targeting PRPK with rocuronium bromide (RocBr) could inhibit the development of cSCC [5].

For patients with localized cSCC, complete surgical resection is indicated, followed by radiotherapy in those with non-resectable tumors [6,7]. Localized cSCC is the most common clinical presentation for cSCC and, therefore, surgical, topical and intralesional approaches are considered the primary types of therapeutic interventions. Importantly, cSCC can also be prevented through therapeutic prevention approaches for pre-cancerous lesions such as actinic keratoses (AKs) and/or cSCC. These options range from topical products such as retinoids, 5-fluoruracil, chemical peels and photodynamic therapy to systemic agents such as acitretin and capecitabine. These interventions have strengths and weakness in efficacy and associated side effects, limiting their application in the general population with the largest cSCC incidence/AK prevalence. The vast majority of patients with increased burden of AKs and cSCCs require combination and rotational therapy. Based on experimental studies, RocBr may have the potential to become another alternative for topical treatment of AK/cSCC, especially in patients of advanced age and with multiple comorbidities. Treatment choice is based on staging, risk stratification and pathological findings, per the National Comprehensive Cancer Network (NCCN) clinical practice guidelines [7]. Other studies have examined alternative therapies, such as epidermal growth factor receptor (EGFR) inhibitors, but are not currently recommended for treatment [6,8,9].

RocBr is an FDA-approved aminosteroid neuromuscular blocking drug administered by injection (Zemoron^®^) and works by decreasing or suppressing the depolarization of acetylcholine on the terminal disc of the muscle cell [10]. A rational approach to utilize RocBr for the targeted non-invasive therapy of cSCC would be a topical skin formulation and, in this work, we have successfully developed a new oil/water emulsion lotion of RocBr for topical skin drug delivery that can be used as an alternative to the intravenous route to potentiate its beneficial effect. Comprehensive physicochemical characterization, such as thermal analysis, imaging by electron microscopy with energy dispersive spectroscopy X-ray spectroscopy, imaging by hot-stage microscopy, molecular fingerprinting by spectroscopy and in vitro properties of RocBr, were conducted in vitro to determine the oil/water partition coefficient that can be used to optimize the formulation, ensuring that the drug is delivered to the skin effectively and achieves the desired therapeutic effect for the first time. Additionally, in vitro cell viability using 2D cell culture of HaCaT and normal human epidermal keratinocytes (NHEK^®^) primary cells was used to determine the toxicity of the RocBr formulations by measuring their proliferation, to determine the safe and effective concentration of RocBr in the topical formulation. Moreover, cell viability studies also provide insights into the permeation behavior of the drug in the skin. Finally, the RocBr lotion was tested for drug permeation and membrane drug retention using Strat-M^®^ synthetic biomimetic membrane with a Franz cell diffusion system. In addition, a reconstructed human epidermis tissue (EpiDerm^®^) was used to evaluate the membrane drug retention and drug diffusion behavior of RocBr through this human skin tissue model. To the authors’ knowledge, this comprehensive and systematic study is the first to report these findings.

## 2. Results

### 2.1. Physicochemical Characterization of Raw Rocuronium Bromide

#### Scanning Electron Microscopy (SEM) and Energy-Dispersive X-ray (EDX) Spectroscopy

**Figure 1:**
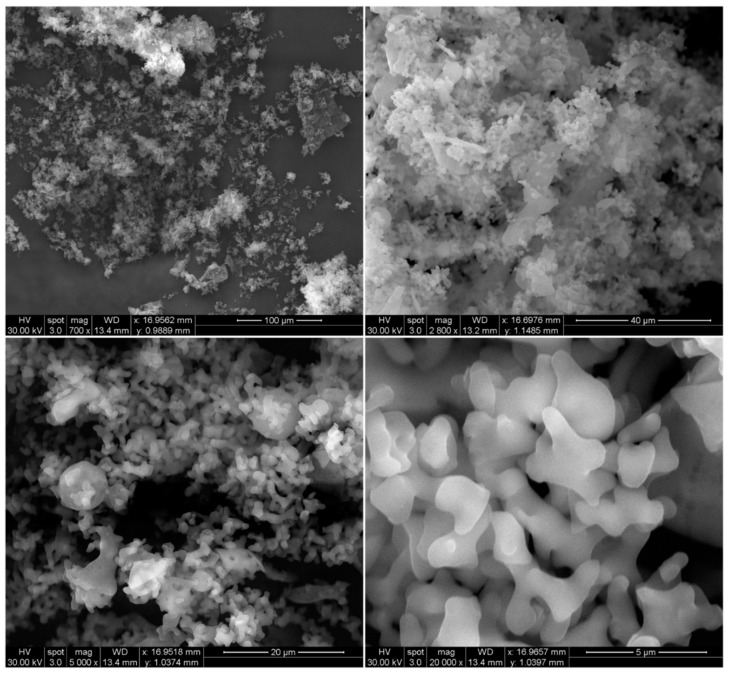
SEM micrographs of RocBr at 700×, 2800×, 5000× and 20,000× resolution.

**Figure 2:**
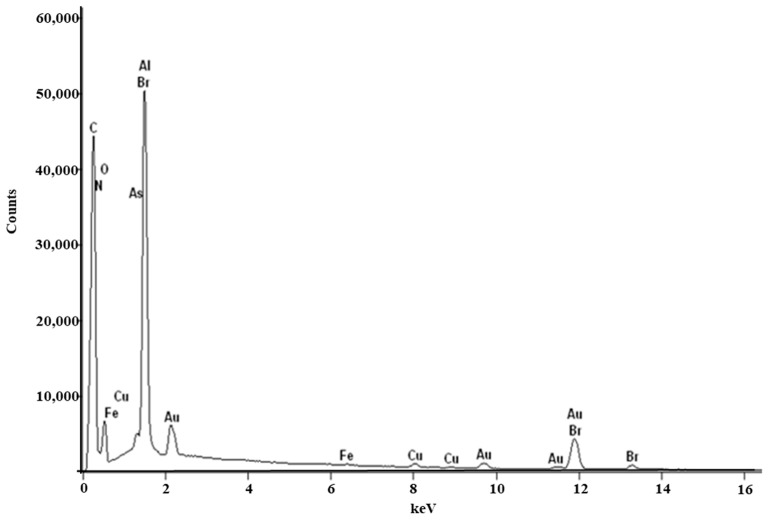
EDX spectra of raw RocBr powder showing characteristic peaks.

Raw RocBr showed an irregular shape (Figure 1) under SEM; as such, it proved difficult to determine its average geometric size. For chemical identification of RocBr (Figure 2), the characteristic Kα lines (peaks) of bromide (Br) were seen at 1.7 keV. The Kα line of carbon (C) was observed at 0.3 keV and the Kα of oxygen (O) and nitrogen (N) were both seen at 0.5 keV. The peaks corresponding to Br, C, O, N and atoms from elemental analysis of RocBr are shown in Figure 2.

### 2.2. X-ray Powder Diffraction (XRPD)

**Figure 3:**
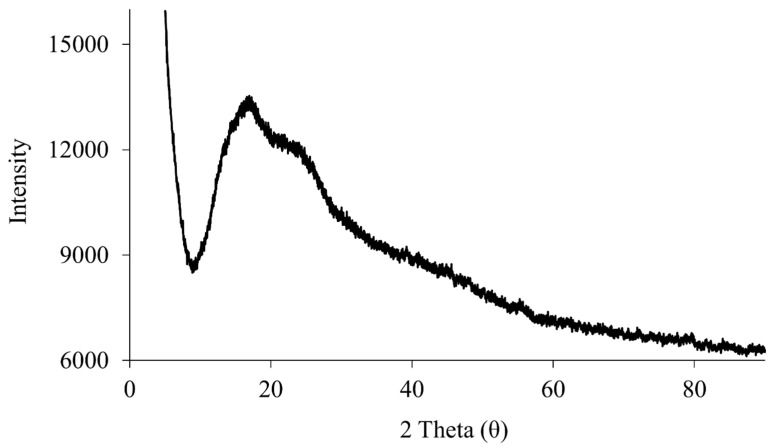
XRPD diffraction patterns of raw RocBr.

XRPD is a non-destructive technique to evaluate the solid-state nature of samples [11]. The XRPD spectrum of raw RocBr (Figure 3) showed only a background hump without any characteristic diffraction peaks, indicating the absence of long-range molecular order, i.e., non-crystalline, amorphous nature of the drug [12].

### 2.3. Differential Scanning Calorimetry (DSC)

**Figure 4:**
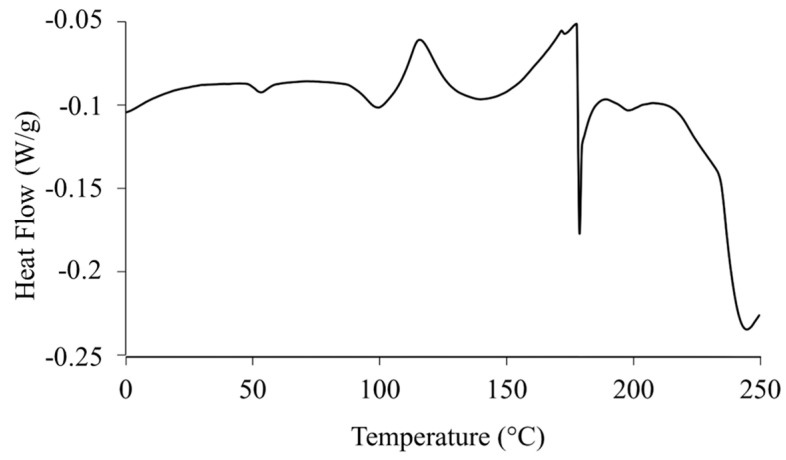
DSC thermogram of raw RocBr.

The DSC thermograms of raw RocBr (Figure 4) exhibited broad endothermic peaks at 53.6 °C, 96.9 °C and 173.2 °C, representing a glass transition (T_g_) step followed by water loss and melting decomposition, respectively. The enthalpy and temperature values are summarized in Table 1. The predicted melting point of RocBr is ~169 °C.

### 2.4. Hot-Stage Microscopy (HSM)

**Figure 5:**
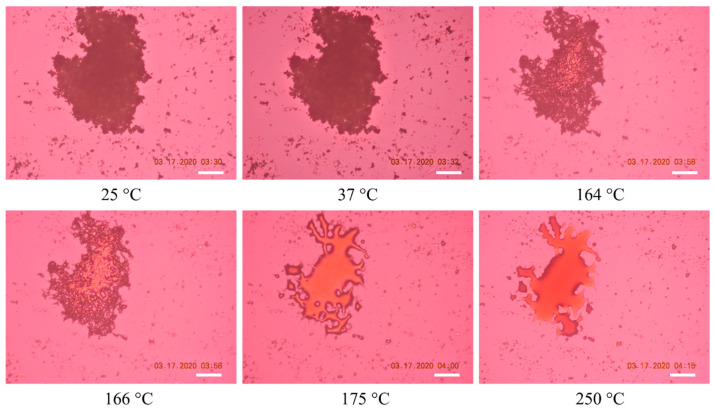
Representative HSM images of raw RocBr. The scale bar represents 100 µm.

Raw RocBr showed irregularly shaped aggregated particles without birefringence, indicating the non-crystalline nature of the solid-state drug, which was consistent with its XRPD diffractogram (Figure 5). At 164–166 °C, raw RocBr solid-state particles started to show a solid-to-liquid phase first-order transition, characteristic of melting, at ~175 °C. As temperature increased, RocBr remained in a liquid state, with possible decomposition shown at 250 °C. The transitions observed during HSM were consistent with DSC thermograms (Figure 4).

### 2.5. Karl Fisher Titration (KFT)

The residual water content of raw RocBr was quantified by Karl Fisher coulometric titration. Raw RocBr powder had an average residual water content of 0.086 % *w*/*w* ± 0.023 (Table 2).

### 2.6. Raman Spectroscopy

**Figure 6:**
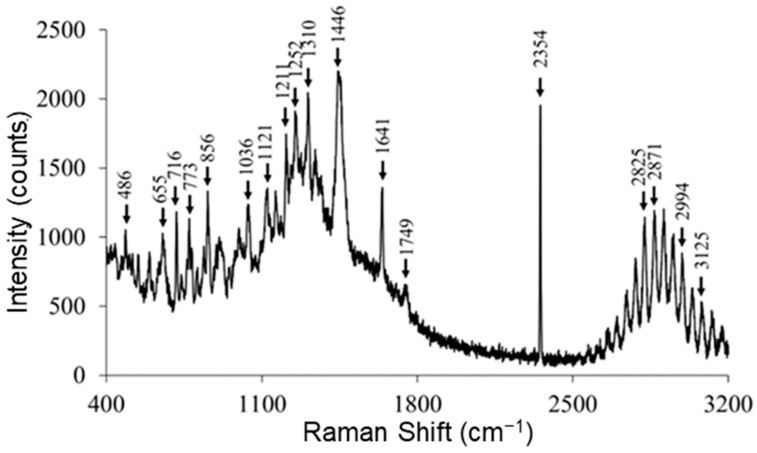
Representative Raman spectra of raw RocBr using 785 nm laser.

The Raman spectrum (Figure 6) of RocBr showed characteristic peaks (cm^−1^) at 3125 (ν O–H, weak), 2994, 2871, 2825 (ν C–H, strong), 2354 (ν C=C, strong), 1749, 1641 (ν C=O, medium), 1446, 1310 (δ CH_2_, medium), 1252, 1211, 1121, 1036, 856, 773, 716 and 655 (ν C–C, medium), as previously reported [13,14,15].

### 2.7. Attenuated Total Reflectance (ATR)–Fourier-Transform Infrared (FTIR) Spectroscopy

**Figure 7:**
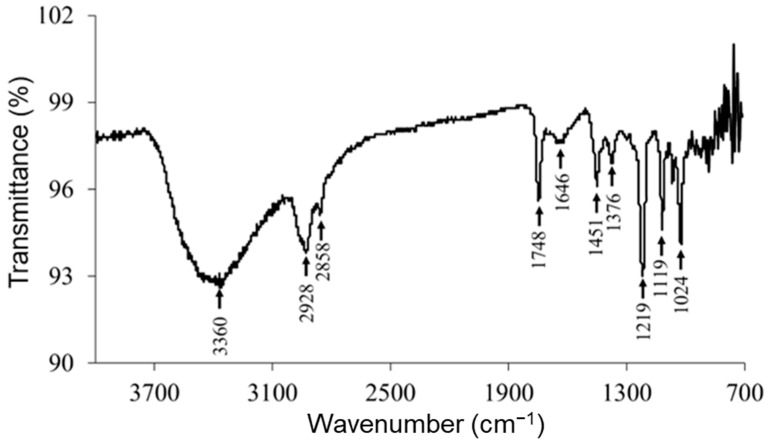
ATR–FTIR spectrum of raw RocBr.

Figure 7 shows the ATR–FTIR spectrum of RocBr. The FTIR spectrum showed characteristic peaks (cm^−1^) at 3360 (ν O–H, phenolic), 2928, 2858 (ν C–H, alkane), 1748 (ν C=O, ester), 1646 (ν C=C, alkene), 1451 (δ O–H, phenolic), 1376, 1219 (ν C–N), 1119 and 1024 (ν C–O, ester), as previously reported [14,15]. The spectral pattern seen in the fingerprint region (<1000 cm^−1^) was consistently observed in raw RocBr.

### 2.8. In Vitro and In Silico Oil/Water Partitioning Coefficient (Log P) of RocBr

The in silico predicted pKa values of RocBr were 7.2 and 14.5 using ChemDraw^TM^ (Ver. 16.0.; Cambridge Soft, Cambridge, MA, USA). The in silico calculated/computed Log P (cLog P) of RocBr was 2.43 using ChemDraw™ Ver. 16.0 (Cambridge Soft, Cambridge, MA, USA) and 1.72 using Swiss ADME (Swiss Institute of Bioinformatics, Switzerland). The experimental partition coefficient of RocBr was measured at pH 7.1 and 12.9. Log P of RocBr at 35 °C ranged from −0.61 to 0.90 and from −0.20 to 0.56 at room temperature (Table 3).

### 2.9. Ultraviolet (UV)/Visible (Vis) Spectroscopy

**Figure 8:**
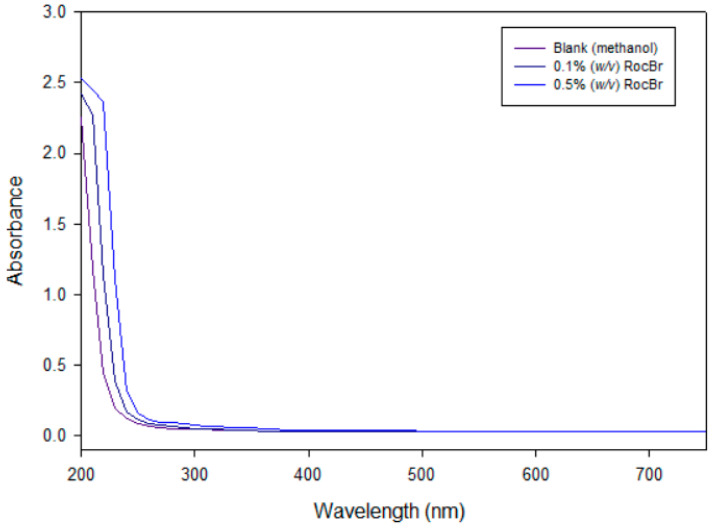
UV/Vis of 0.1% and 0.5% (*w*/*v*) RocBr in HPLC-grade methanol, as compared to HPLC-grade methanol alone over a range of 200 nm to 750 nm.

An absorption below wavelength of 200–250 nm (UVA) with a lambda maximum was observed in the UVA region at 210 nm, in both 0.1% (*w*/*v*) and 0.5% (*w*/*v*) RocBr solutions, when compared to methanol as a blank (Figure 8).

### 2.10. High Performance Liquid Chromatography (HPLC) Analysis

**Figure 9:**
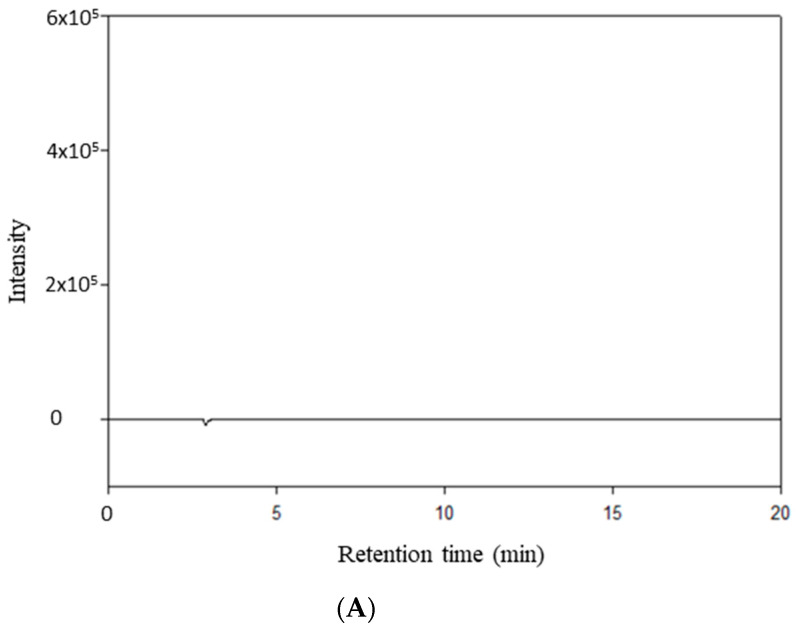

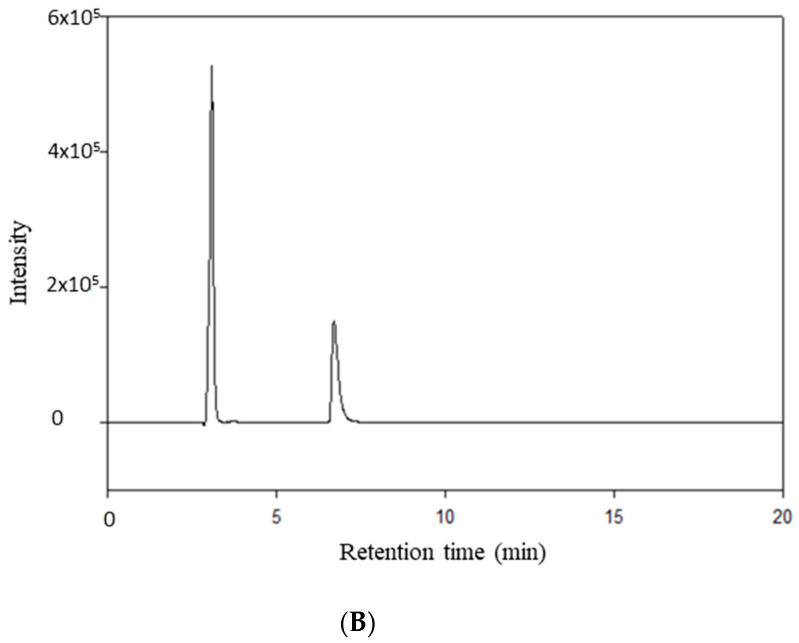
HPLC chromatogram of (**A**) HPLC-grade acetonitrile; (**B**) RocBr.

RocBr showed an average retention time of 7.02 ± 0.04 min (Figure 9B) when compared with acetonitrile (Figure 9A).

### 2.11. In Silico ADME Prediction

The Lipinski Rule of Five is a widely used guideline in drug design and development that predicts a compound’s likelihood of being bioavailable. A set of rules known as the Lipinski Rule of Five is frequently applied in the drug discovery process to assess the possibility that a drug candidate would be successful in terms of being absorbed by the body. The rule is founded on the idea that substances which have particular physicochemical characteristics are more likely to have good oral bioavailability, which refers to a drug’s capacity to enter the bloodstream.

A quaternary ammonium chemical called rocuronium bromide was first employed as a skeletal muscle relaxant. Its physicochemical characteristics are crucial to its active ingredient cutaneous and transdermal distribution. RocBr has a 610.78 g/mol molecular weight, which is a high value. Due to their large size, high-MW drugs are usually considered to have poor skin permeability. However, because rocuronium bromide is a charged chemical, ions could partner with skin lipids, which could increase skin penetration. Important factors in predicting skin permeation include the quantity of H-bond donors (NHD) and acceptors (NHA). With one H-bond donor and four H-bond acceptors, RocBr may be able to form a hydrogen bond with the skin molecules, improving its permeability. In general, RocBr physicochemical characteristics indicate that, depending on the particular skin conditions and formulation employed, it may have moderate-to-high skin permeability.

### 2.12. In Vitro Cell Dose–Response Assay in 2D Cell Culture

**Figure 10:**
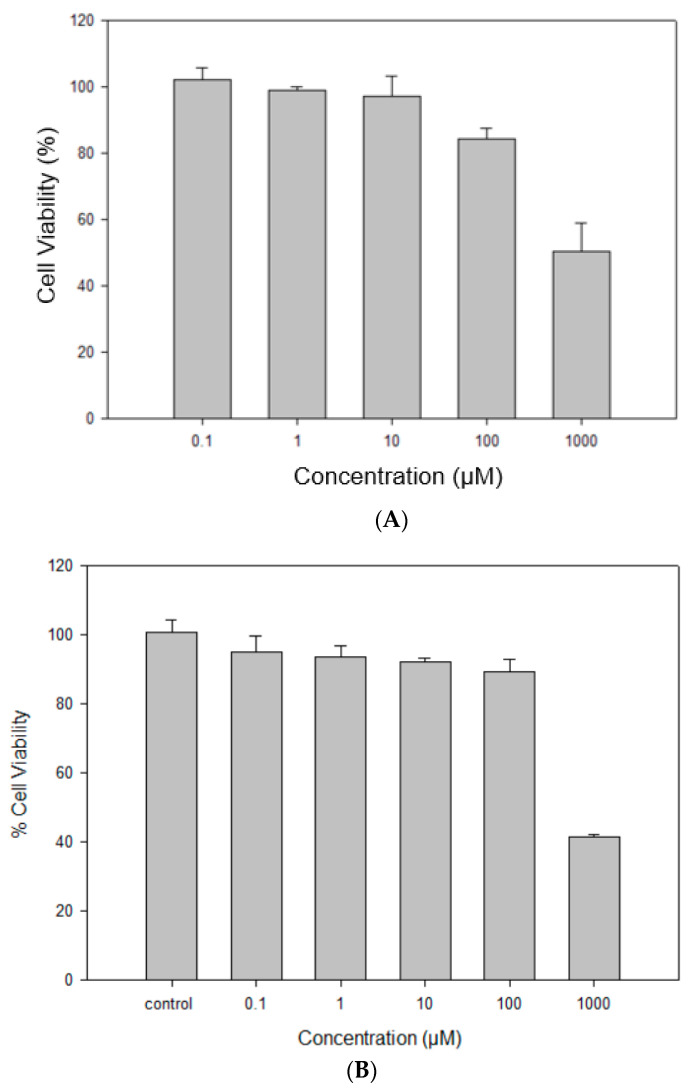
In vitro cell viability of raw RocBr using (**A**) human transformed keratinocytes (HaCaT), (**B**) primary normal human epidermal keratinocytes (NHEK).

RocBr did not show any significant reduction in cell viability in either HaCaT or NHEK cells after 48 h of exposure to varying dose concentrations of drug, comparing experimental groups to control groups without RocBr (Figure 10A,B). The viability of NHEK primary cells was ~100% at all drug dose concentrations except 1000 µM. At 1000 µM drug concentration, the NHEK primary cell viability decreased significantly (*p* < 0.0001) compared to the other concentrations tested. The viability of HaCaT skin cells was ~100% when treated with RocBr at dose concentrations of 0.01 µM, 1 µM or 10 µM. The viability of HaCaT cells decreased significantly at 100 µM (*p* < 0.05) and 1000 µM (*p* < 0.0001) drug concentrations, compared to the lower concentrations tested.

### 2.13. In Vitro Permeation of RocBr through Strat-M^®^ Transdermal Diffusion Membrane

A Strat-M^®^ transdermal diffusion membrane was utilized to evaluate in vitro diffusion and permeation of RocBr formulations. The analysis was conducted with 1% (*w*/*v*) RocBr lotion and 1% (*w*/*v*) RocBr solution. RocBr in 1% (*w*/*v*) solution and lotion exhibited no measurable permeation through/diffusion out of the Strat-M^®^ membrane at all the timepoints. As expected, RocBr lotion showed significantly (*p* < 0.0001) higher retention (953.9 ± 17.9 µg) on the membrane than that of solution (768.3 ± 12.6 µg) (Table 4).

**Figure 11:**
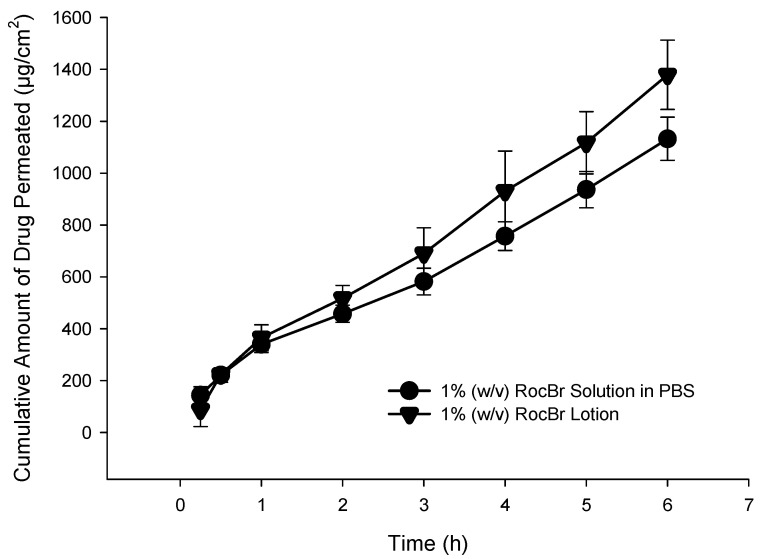
In vitro EpiDerm^TM^ permeation profile of RocBr. 1% (*w*/*v*) PBS (pH = 7.4) solution and 1% (*w*/*v*) RocBr lotion.

Diffusion through the 3D cell model is relevant, as it helps to predict RocBr skin depth penetration capacity and drug accumulation in the human tissue, which are essential for topical delivery for skin carcinogenesis treatment. Epiderm™ was used to study drug permeation of 1% (*w*/*v*) RocBr lotion and solution over the course of 6 h (Figure 11). Flux (J) value from 1% (*w*/*v*) lotion was lower than that of drug solution. On the other hand, RocBr lotion (29.2 ± 6.76 µg) showed less retention than RocBr solution (Table 5).

## 3. Discussion

RocBr is a steroidal neuromuscular blocking agent and is commonly used as a muscle relaxant during surgery or mechanical ventilation [16]. Recently, RocBr was identified as an inhibitor of PRPK, attenuating the development of solar-simulated light-induced cSCC and expression of proliferation and oncogenesis markers in SKH1 hairless mice [5]. A preformulation study to understand the physicochemical and solid-state nature of the drug is important. Thus, an in-depth physicochemical characterization and solid-state analysis of raw RocBr was conducted. Comprehensive physicochemical characterization—thermal analysis, imaging by electron microscopy with energy dispersive spectroscopy X-ray spectroscopy, imaging by hot-stage microscopy, molecular fingerprinting by spectroscopy and in vitro properties of RocBr—was conducted and reported for the first time. RocBr powder appears to be non-crystalline at room and biological temperatures, as indicated by the lack of sharp diffraction peaks which are characteristic of long-range molecular order in crystalline powders in XRPD, the lack of crystalline birefringence in HSM and the exothermic peak indicative of a disorder-to-order solid-state first-order phase transition followed by melting in DSC, which is also a first-order phase transition. RocBr powder has a measurable first-order phase transition of melting at a high temperature well above 100 °C. The very low residual water content of the RocBr powder is consistent with hydrophobicity. In addition, we have successfully developed, for the first time, a RocBr lotion with biocompatible excipients that are commonly used in the cosmetics industry.

In vitro cell viability was evaluated with 2D human cell culture of HaCaT skin cells and 2D normal human epidermal keratinocytes (NHEK^®^). Keratinocytes play a vital role in providing functions and structure to the skin [17]. Normal human epidermal keratinocytes (NHEK) are primary cells collected from the epidermis of adult donors, which have been widely used as a model for inflammatory skin diseases and skin responses to ultraviolet radiation or oxidative stress [18,19,20,21,22]. HaCaT is a spontaneously immortalized keratinocyte line derived from adult trunk skin and has been widely used as a reliable in vitro model for studies of epidermal architecture, inflammatory responses and skin metabolism [23,24,25,26]. In addition, HaCaT cells have p53 mutations that are characteristic of cutaneous squamous cell carcinomas and, thus, are considered as a relevant model for analyzing skin cancer development [27]. In vitro toxicity of RocBr on human skin cells was successfully demonstrated over a wide drug concentration dose range using 2D cell culture. This was evident for both the HaCaT human keratinocyte cell line and NHEK primary cells. The findings indicate that the viability of NHEK primary cells and HaCaT cells remained high at all tested drug dose concentrations, except for the highest concentration (1000 µM). At this concentration, the NHEK primary cell viability decreased significantly compared to the other concentrations tested. The significant difference in viability at the highest dose concentration compared to the others indicates that there is likely a dose-dependent effect on cell viability. The high cell viability observed in this study at the lower drug concentrations indicates that the drug may be safe to use at therapeutic doses.

In vitro oil/water partition coefficient at two different temperatures (representing ambient and skin temperature) were successfully completed and reported for the first time. In vitro Log P was measured under various conditions and was found to be ~0, which is consistent with amphiphilic drugs which demonstrate near-equal partitioning of drug molecules into both the hydrophobic octanol phase and hydrophilic aqueous phase.

In vitro models are very important for predicting drug permeation through skin in the early stage of formulation development. Strat-M^®^ membrane is a synthetic model designed to mimic the structure and lipid composition of human skin, which has been widely used to evaluate topical formulations [28,29,30,31,32] and screen between different formulations for their optimization. While the Strat-M^®^ membrane can be used for studying drug permeation across the skin, it is not identical to the complex structure and properties of the skin. Another in vitro model, EpiDerm^®^, was used as a three-dimensional tissue model, consisting of multiple layers of normal human epidermal keratinocytes on inserts [33]. EpiDerm^®^ model exhibits similar morphology, lipid profile, metabolic activity and barrier functions of normal human skin and, thus, it has been used for assessing skin irritancy, phototoxicity and drug transport [34]. The new RocBr lotion formulation was successfully tested for drug permeation and membrane drug retention using Strat-M^®^ synthetic biomimetic membrane with the in vitro Franz cell diffusion system. No measurable drug flux was observed for RocBr lotion nor RocBr solution using the in vitro Strat-M^®^ synthetic biomimetic membrane/Franz cell diffusion system. RocBr lotion provided significantly (*p* < 0.0001) higher retention in the Strat-M^®^ membrane compared to the RocBr solution using the in vitro Strat-M^®^ synthetic biomimetic membrane/Franz cell diffusion system. This demonstrates that both formulations are suitable for targeted skin drug delivery with relatively high tissue retention, with the lotion being superior to the solution under these conditions.

In addition to the in vitro Strat-M^®^ synthetic biomimetic membrane/Franz cell diffusion system, EpiDerm^®^ 3D human tissue was successfully used to evaluate the membrane drug retention and drug diffusion behavior of RocBr in vitro. Both RocBr lotion and solution formulations demonstrated measurable drug flux and membrane retention. However, RocBr solution demonstrated relatively higher values of both flux and tissue membrane retention than the RocBr lotion when using EpiDerm^®^ 3D human tissue. One advantage of using EpiDerm^®^ tissue for drug permeation studies is its close resemblance to human skin, which can provide more accurate and representative results compared to synthetic membranes such as Strat-M^®^. However, EpiDerm^TM^ tissue typically has a thickness of 0.3 to 0.4 mm, which can limit its use for studying the permeation of drugs with regard to deeper skin penetration. Another plausible explanation for the difference in results in both Strat-M^®^ and EpiDerm^TM^ is the potential for RocBr skin penetration. Molecular modeling showed how RocBr may have poor penetration given its molecular weight being >500 g/mol, despite its low Log P, as shown in Table 3. Furthermore, other factors which could have impacted penetration are pH of formulation, hydration of membranes and concentration of dissolved drug.

This again demonstrates that both formulations are suitable for targeted skin drug delivery with relatively high tissue retention, with the lotion being superior to the solution under these conditions.

## 4. Materials and Methods 

### 4.1. Materials

**Figure 12:**
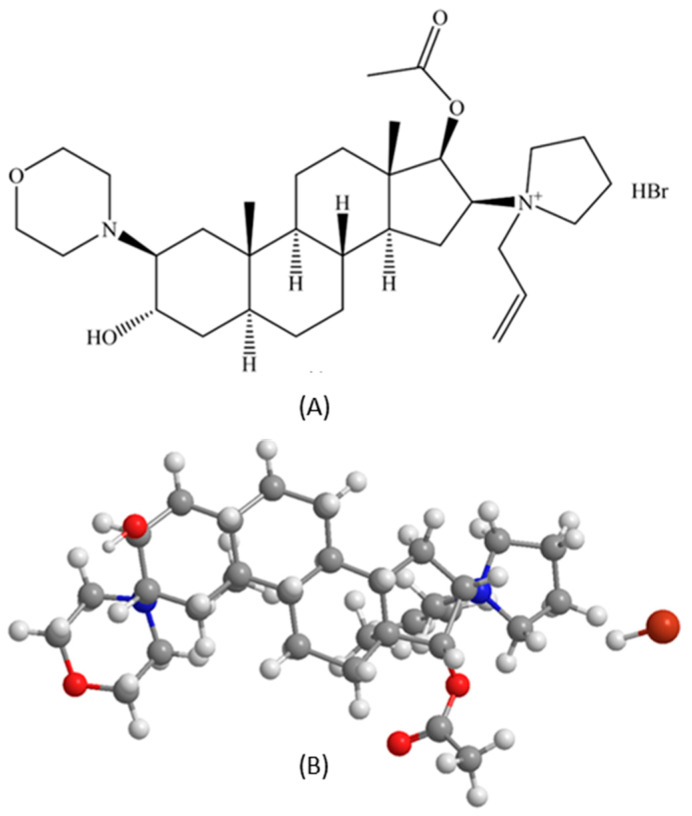
Rocuronium bromide (RocBr): (**A**) chemical structure (**B**) 3D ball-and-stick chemical structure (ChemDraw^TM^ Ver. 16.0.; Cambridge Soft, Cambridge, MA, USA).

Rocuronium bromide (purity 99%, molecular weight 529.79 g/mol, C_32_H_53_N_2_O_4_^+^Br^−^) was purchased from MuseChem (Fairfield, NJ, USA), with structure as shown in Figure 12 (ChemDraw^TM^ Ver. 16.0.; Cambridge Soft, Cambridge, MA, USA). Sodium bromide (purity 99%), tetramethylammonium hydroxide pentahydrate (purity > 97%), phosphoric acid for HPLC (purity 85–90%) and Hydranal^®^-Coulomat AD and resazurin sodium salt were purchased from Sigma-Aldrich (St. Louis, MO, USA). Sodium chloride (purity 99%, salt, crystal, reagent, A.C.S) was purchased from Spectrum Chemical MFG Corp. (Gardena, CA, USA). The digital thermometer/hygrometer LSCs were purchased from Veanic (Shenzhenshi Aoyu Keji Co., Ltd., Shenzhen, China). The 0.2 µm nylon membranes (25 mm) were purchased from VWR (Radnor, PA, USA).

The human keratinocyte immortal cell line (HaCaT, AddexBio^®^ T0020001) was purchased from AddexBio (San Diego, CA, USA). The Dulbecco’s modified Eagle’s medium (DMEM), optimized 1X was obtained from AddexBio (San Diego, CA, USA). Fetal bovine serum (FBS), Pen-Strep (5000 U/mL) and Fungizone^®^ were obtained from Gibco^TM^ Life Technologies (Thermo Fisher Inc, Waltham, MA, USA). Normal human epidermal keratinocytes (NHEK^®^), which are primary adult cells, and their growth media (NHEK-GM^®^) were both purchased from MatTek Life Sciences (Ashland, MA, USA). EpiDerm^TM^, a 3D tissue model consisting of normal human-derived epidermal keratinocytes, and its EpiDerm^TM^ special growth medium were purchased from MatTek Life Sciences (Ashland, MA, USA). The Strat-M^®^ membrane (Transdermal Diffusion Test Model, 47 mm) was purchased from Millipore Sigma (Danvers, MA, USA).

### 4.2. Preparation of the Topical Oil/Water Emulsion Formulation

The rocuronium bromide (RocBr) 1% (*w*/*v*) oil/water emulsion lotion was prepared by the Dr. Ann Bode lab at The Hormel Institute at the University of Minnesota (Austin, MN, USA) [35]. Briefly, oil-in-water lotion of RocBr was prepared by mixing Phase A and B at 70 °C. All ingredients in Phase A and B were melted at 75–80 °C before mixing. Phase A comprised 0.05 g sodium salt of ethylenediamine tetra-acetic acid (nonallergenic preservative and stabilizer), 3 mL 1,3-butylene glycol (humectant), 4 mL glycerin (humectant), 2 mL pentylene glycol (skin conditioning agent) and distilled water to prepare 100 mL; phase B comprised 1.2 g cetyl alcohol (emulsion stabilizer), 1.5 g glyceryl stearate (emulsifying agent) and 8 mL hydrogenated polydecene (skin conditioning agent). RocBr powder was slowly added during continuous mixing of phase A and phase B at 70 °C and then the mixture was allowed to cool down to 30–35 °C. RocBr lotion was stored at 4 °C until further evaluation.

### 4.3. Scanning Electron Microscopy (SEM) and Energy-Dispersive X-ray (EDX) Spectroscopy

Surface morphology of raw RocBr powder was visualized under scanning electron microscopy (SEM) (FEI, Brno, Czech Republic). The sample was mounted on the stub with double-sided adhesive carbon tape (TedPatella, Inc., Redding, CA, USA) and coated with a 7 nm thin film of gold palladium alloy under argon plasma in the Anatech Hummer 6.2 sputtering system (Union City, CA, USA). SEM micrographs were captured under 30 kV accelerating voltage at a working distance of approximately 9–12 mm. Mean size, standard deviation and size range of the particles were determined digitally in SEM micrographs using SigmaScan^TM^ Pro 5.0.0 (Systat, Inc., San Jose, CA, USA). At least 60 particles were measured in the representative micrographs of raw RocBr powder at 400–5000× magnification.

EDX for elemental fingerprinting was performed on raw RocBr powder with Thermo Noran System Six (Thermo Scientific, Waltham, MA, USA) at an accumulation voltage of 30 keV. The spot size was increased until a dead time of 20–30 s was reached.

### 4.4. X-ray Powder Diffraction (XRPD)

The crystalline nature of raw RocBr powder was determined by a PANalytical X’pert diffractometer (PANalytical Inc., Westborough, MA, USA) equipped with a programmable incident beam slit and an X’celerator detector. The X-ray radiation used was Ni-filtered Cu Kα (45 kV, 40 Ma and λ = 1.5444 Å). Measurements were taken between 5° and 89.9° (2θ) with a scan rate of 2.00°/min. The powder samples were loaded on a zero-background silicon sample holder.

### 4.5. Differential Scanning Calorimetry (DSC)

A TA Q1000 differential scanning calorimeter, with autosampler and RSC autocooling system (TA Instruments, New Castle, DE, USA), was used to analyze the thermal transition of raw RocBr powder. Approximately 1–3 mg of raw RocBr were weighed and hermetically sealed in a DSC pan (TA Instruments, New Castle, DE, USA). An empty hermetically sealed aluminum pan was used as a reference. UHP nitrogen gas was used as the purging gas at a flow rate of 40 mL/min. The samples were heated from 0.00 °C to 250.00 °C at a scanning rate of 5.00 °C/min. All measurements were conducted in triplicate.

### 4.6. Hot-Stage Microscopy (HSM)

Thermal changes of raw RocBr powder during heating were observed under a Leica DMLP cross-polarized microscope (Wetzlar, Germany) equipped with a Mettler FP 80 central processor heating unit and a Mettler FP82 hot stage (Columbus, OH, USA). Raw RocBr powder was mounted onto a glass slide and heated from 25 °C to 250 °C at a heating rate of 5.00 °C/min. The images were digitally captured with a Nikon Coolpix 8800 digital camera (Nikon, Tokyo, Japan) under 10× optical objective and 10× digital zoom.

### 4.7. Karl Fisher Titration (KFT)

Coulometric KFT was utilized to determine the residual water content of raw RocBr powder. Briefly, RocBr was dissolved in anhydrous methanol at 1 μg/mL (0.1% *w*/*v*) and 1 mL of solution was added to the titration cell containing Hydranal^®^ Coulomat AD reagent in a TitroLine 750 trace titrator (SI Analytics, Weilheim, Germany).

### 4.8. Raman Spectroscopy

Raman spectra for molecular fingerprinting of raw RocBr powder were obtained using Renishaw inVia Reflex (Gloucestershire, United Kingdom) at exciting laser wavelength of 785 nm, under Leica DM2700 optical microscope (Wetzlar, Germany) at 20× magnification. The scans were obtained with 1% of laser power and 10 s exposure time. Baseline correction was made in the spectra prior to analysis with Renishaw WiRE 3.4 software.

### 4.9. Attenuated Total Reflectance (ATR)–Fourier-Transform Infrared (FTIR) Spectroscopy

ATR–FTIR spectra for molecular fingerprinting of raw RocBr powder were obtained using a Nicolet Avatar 360 FTIR spectrometer (Varian, Inc.,Palo Alto, CA, USA) to determine the molecular fingerprint and presence of functional groups of RocBr. Each spectrum was collected over the wavenumber range of 4000–700 cm^−1^ after 32 scans at a resolution of 2 cm^−1^. A background spectrum was obtained under the same conditions. EZ-OMNIC software version 7.3 was used to acquire and analyze the spectra.

### 4.10. Ultraviolet (UV)/Visible (Vis) Spectroscopy

UV/Vis spectra of raw RocBr were obtained with Molecular Devices^®^ SpectraMax^®^ M3 Multi-Mode Microplate Reader (Sunnyvale, CA, USA) from 200 nm to 750 nm. RocBr was dissolved in methanol at 0.1% and 0.5% (*w*/*v*) and analyzed in a 96-well plate.

### 4.11. High Performance Liquid Chromatography (HPLC) Analysis

The HPLC analysis was performed on a Shimadzu LC-2010A HT liquid chromatograph (Torrance, CA, USA) coupled with a UV–Vis dual wavelength detector and a Luna Silica, 5 µm column (250 mm × 4.6 mm) (Phenomenex, Torrance, CA, USA). RocBr was detected at 210 nm. The mobile phase was 60:40 (*v*/*v*) acetonitrile:tetramethylammonium hydroxide pentahydrate (0.025M), with pH adjusted to approximately 7.4 with 1:9 (*v*/*v*) phosphoric acid:acetonitrile solution. The flow rate was set to 1.0 mL/min and injection volume was 10 µL, as previously reported [36,37]. The retention time for RocBr was ~7 min. Drug concentration was determined with a five-point standard curve (0.03125 mg/mL to 1 mg/mL, R^2^ = 0.9998). Standards were prepared by serial dilution of RocBr bulk solution with acetonitrile and stored at 4 °C, protected from light.

### 4.12. In Vitro and In Silico Oil/Water Partition Coefficient (Log P) of RocBr

For in vitro measurements, 3 mg of RocBr powder was added to an amber glass vial containing equal volume (1.5 mL) of 1-octanol and phosphate buffered saline (PBS, 1×, pH 7.4) to make a 1 mg/mL solution. The pH was adjusted to 7.1 and 12.9 with 0.1 M hydrochloric acid (HCl) solution and 0.1 M sodium hydroxide (NaOH) solution, respectively. Log P of RocBr was determined using Equation (1):

Log P = Log{[RocBr]_oil_/[RocBr]_water_} (1)

Two temperatures—ambient room temperature and 35 °C, which is the widely reported and generally accepted average human skin temperature—were used. The vials were rotated for 24 h and then left undisturbed in a vertical position for phase separation for the next 24 h. A volume of 200 µL of the organic and aqueous layer were sampled very carefully without disturbing the interface and analyzed using the HPLC method described above.

For in silico predictive modeling, ChemDraw™ Ver. 16.0 (Cambridge Soft, Cambridge, MA, USA) and Swiss ADME (Swiss Institute of Bioinformatics, Switzerland) web server were used. The purpose of molecular modeling was to have theoretical values for the physicochemical properties, lipophilicity and water solubility of rocuronium bromide. Theoretical Log P was compared to the in vitro results.

### 4.13. In Silico ADME Prediction

Lipinski’s Rule of Five and skin permeation RocBr were all tested utilizing the SwissADME web server.

### 4.14. In Vitro Cell Viability by 2D Cell Culture of a Human Skin Cell Line and Human Primary Skin Cells

The cell viability of RocBr on human epidermis was evaluated with the HaCaT human keratinocyte immortal cell line and NHEK (normal human epidermal keratinocyte) primary cells as 2D cell culture. The HaCaT cells were grown in Dulbecco’s modified Eagle’s medium (DMEM, Optimized 1X), supplemented with 10% (*v*/*v*) fetal bovine serum (FBS) and Pen-Strep (100 units/mL penicillin, 100 µg/mL) in a humidified incubator at 37 °C and 5% CO_2_. NHEK primary cells were grown in the medium provided by MatTek (Ashland, MA, USA) in a humidified incubator at 37 °C and 5% CO_2_.

HaCaT and NHEK cells were seeded into 96-black well plates at 5000 cells in 100 µL medium per well. After 48 h, cells were exposed to RocBr at concentrations of 1000 µM, 100 µM, 10 µM, 1 µM or 0.1 µM. Drug solutions were prepared by dissolving raw RocBr powder in 1 mL of HPLC-grade ethanol (EtOH) and diluted with 9 mL of growth media. A volume of 100 µL of drug solution was added to each well. After 48 h, 20 µL of 20 µM resazurin sodium salt was added to each well and incubated for 4 h. Fluorescence intensity of resofurin was detected at 544 nm (excitation) and 590 nm (emission) using the Molecular Devices^®^ SpectraMax^®^ M3 Multi-Mode Microplate Reader (Sunnyvale, CA, USA). The relative viability of the cells was calculated by Equation (2) as follows:

(2) $$\mathrm{Relative}\mathrm{Viability}\%=\frac{\mathrm{Sample}\mathrm{flourescence}\mathrm{intensity}}{\mathrm{Control}\mathrm{flourescece}\mathrm{intensity}}\times 100\%$$

### 4.15. In Vitro Permeation of RocBr Using Strat-M^®^ Synthetic Biomimetic Membrane and Franz Cell Diffusion System

Strat-M^®^ synthetic membrane (Sigma Aldrich, St. Louis, MO, USA) inside a glass Franz Cell Diffusion system (Permegear, Hellertown, PA, USA) was used to study the drug membrane permeation and retention of RocBr from RocBr solution (1% *w*/*v* RocBr solution prepared in PBS, pH 7.4, 200 µL) and lotion formulations. The membrane diameter available for diffusion was 5.0 cm. The RocBr formulation was prepared as described in the methods section. PBS (pH 7.4) mixed with 10% (*v*/*v*) ethanol was used as the receptor medium. The receptor compartment was filled with 5 mL of medium and maintained at 35 °C, the well-reported and generally accepted average human skin temperature, in a reciprocal shaking bath model 25 (Thermo Fisher Scientific, Fair Lawn, NJ, USA) at 30 oscillations/minute. A volume of 200 μL of RocBr solution and RocBr lotion were added onto the membrane and the effective diffusion area was 0.64 cm^2^. At predetermined time intervals, 200 µL of the receptor medium was sampled and replaced with an equal volume of fresh medium. The flux at steady-state (J) was estimated as the slope of the linear regression analysis of the linear portion of the permeation curve. Lag time (Lt) was defined as the time intercept of the steady-state region of the permeation curve (i.e., x-intercept) [38]. The cumulative drug permeation and drug retention on the membrane were quantified with the HPLC method described in the Methods section.

### 4.16. In Vitro Permeation of RocBr by Using 3D Normal Human-Derived Epidermal Keratinocytes (EpiDerm^TM^) and MatTek Permeation Device

Following Mat-Tek’s protocol [39] and the MatTek Permeation Device (MatTek, Ashland, MA, USA), EpiDerm^TM^ samples were placed in tissue culture inserts and transferred onto a 6-well cell culture plate. Each well was pre-filled with 1 mL of Dulbecco’s phosphate buffered saline without calcium chloride (CaCl_2_) or magnesium chloride (MgCl_2_) (Mat-Tek, Ashland, MA, USA). The plate was placed in a reciprocal shaking bath model 25 (Thermo Fisher Scientific, Fair Lawn, NJ, USA) at 30 oscillations per minute and maintained at 35 °C ± 0.05 °C, the well-reported and generally accepted average human skin temperature. A volume of 400 μL of RocBr lotion was added onto the EpiDerm^TM^ and the effective diffusion area was 0.256 cm^2^. The same HPLC method described earlier was used.

### 4.17. Statistical Analysis

The data are presented as the mean ± standard deviation, derived from three independent experiments (n = 3). The statistical difference between the results of cell viability and drug retention were compared by one-way analysis of variance (ANOVA) with Tukey’s post hoc test for comparisons (Prism 9.0, GraphPad Software, San Diego, CA, USA). In all cases, the *p* values of 0.05 or less were considered significant.

## 5. Conclusions

In conclusion, this systematic and comprehensive study reports several new findings for the first time. These include comprehensive physicochemical characterization, thermal analysis, imaging by electron microscopy with energy dispersive spectroscopy X-ray spectroscopy, imaging by hot-stage microscopy, molecular fingerprinting by spectroscopy and in vitro properties of RocBr. These in vitro properties include oil/water partition coefficient at two different temperatures, showing equal distribution of drug molecules in the octanol and water phases, consistent which drug amphilicity, Also observed were low toxicity in 2D human skin cell culture of the HaCaT human keratinocyte cell line over a wide drug concentration, low toxicity in 2D normal human epidermal keratinocytes (NHEK^®^) primary cells over a wide drug concentration, drug permeation and membrane retention using Strat-M^®^ synthetic biomimetic membrane and drug permeation and membrane drug retention using EpiDerm ^TM^ human skin tissue.

A topical oil/water emulsion lotion formulation was developed and evaluated. The in vitro permeation behavior of RocBr from its lotion formulation was quantified with Strat-M^®^ synthetic biomimetic membrane and EpiDerm^TM^ 3D human skin tissue. Significant membrane retention of RocBr drug was evident and more retention was obtained with the lotion formulation compared with the solution. Drug penetration of RocBr lotion was evaluated in two in vitro models, using STRAT-M^®^ synthetic biomimetic membrane/Franz cell diffusion system and Epiderm^®^ human tissue. Drug retention in the membrane was quantifiable and relatively high. Drug flux out of the membrane was relatively low, which is favorable for local skin delivery to treat non-melanoma skin cancer while minimizing systemic exposure. Clinical evaluation for treating non-melanoma skin cancer would be needed to assess clinical application.

## Figures and Tables

**Table 1:** DSC thermal analysis of RocBr (n = 3, mean ± standard deviation).

Sample	Peak 1		Peak 2		Peak 3	
	T_peak_ (°C)	Enthalpy (J/g)	T_peak_ (°C)	Enthalpy (J/g)	T_peak_ (°C)	Enthalpy (J/g)
Raw RocBr	53.6 ± 0.2	0.3 ± 0.1	96.9 ± 3.8	5.6 ± 0.8	173.2 ± 8.3	4.0 ± 0.3

**Table 2:** Residual water content in raw RocBr powder quantified by KFT (n = 3, mean ± standard deviation).

Sample Identification	Residual Water Content (% *w*/*w*)
RocBr1	0.065
RocBr2	0.084
RocBr3	0.110
Average ± SD	0.086 ± 0.023

**Table 3:** Summary of partition coefficient (Log P) for RocBr at various pH values (n = 3, mean ± standard deviation).

Experimental (Log P)	Average ± SD
At 35 °C, pH = 7.1	−0.61 ± 1.10
At Room temperature/ambient temperature, pH = 7.1	−0.20 ± 0.30
**Experimental (Log P)**	**Avg. Log P ± SD**
At 35 °C, pH = 12.9	0.90 ± 0.15
At Room temperature/ambient temperature, pH = 12.9	0.56 ± 0.48
**Predicted (cLog P)**	**Predicted Value**
ChemDraw Version 16.0	2.43
Swiss ADME	1.72

**Table 4:** In vitro skin permeation parameters of RocBr 1% (*w*/*v*) lotion and 1% (*w*/*v*) solution through Strat-M^®^ transdermal diffusion membrane (n = 3, mean ± standard deviation).

Sample	Flux (µg/cm^2^/h)	Lag Time (h)	Drug Retention (µg)
1% (*w*/*v*) solution in PBS (pH = 7.4)		-	768.3 ± 12.6
1% (*w*/*v*) RocBr lotion	-	-	953.9 ± 17.9

**Table 5:** In vitro skin permeation parameters of RocBr 1% (*w*/*v*) lotion and 1% (*w*/*v*) solution through EpiDerm^TM^ 3D normal human-derived epidermal keratinocytes (n = 3, mean ± standard deviation).

Sample	Flux (µg/cm^2^/h)	Lag Time (h)	Drug Retention (µg)
1% (*w*/*v*) solution in PBS (pH = 7.4)	177.2 ± 9.25	-	63.5 ± 19.8
1% (*w*/*v*) RocBr lotion	112.0 ± 12.0	-	29.2 ± 6.76

## Data Availability

The data presented in this study are available on request from the Corresponding Author.
